# Reliability assessment between clinical attachment loss and alveolar bone level in dental radiographs

**DOI:** 10.1002/cre2.324

**Published:** 2020-09-12

**Authors:** Fathima Fazrina Farook, Hussah Alodwene, Rasha Alharbi, Meral Alyami, Amjad Alshahrani, Duaa Almohammadi, Bothinah Alnasyan, Wael Aboelmaaty

**Affiliations:** ^1^ College of Dentistry King Saud bin Abdulaziz University for Health Sciences Riyadh Saudi Arabia; ^2^ King Abdullah International Medical Research Center Riyadh Saudi Arabia; ^3^ Oral Radiology and Diagnostic Sciences, Faculty of Dentistry, Mansoura University Egypt

## Abstract

**Background:**

The clinical attachment level (CAL) and radiographically assessed bone levels are used to assess the loss of periodontal tissue support in periodontitis, a chronic, multifactorial inflammatory disease of the periodontium. However, few studies have been done to study the relationship between these two parameters. According to our knowledge, this is the first study investigating the relationship between the two measurements using intraclass correlation analysis.

**Aim:**

The aim of the study is to investigate the relationship between CAL and radiographically assessed bone level in teeth affected with periodontitis.

**Methods:**

A retrospective cross‐sectional study was conducted by selecting a sample of 880 periodontal sites in 104 periodontitis patients, aged 25–60 years. CAL and peri‐apical radiographs of the selected sites were obtained from the computerized patient records. The distance from the cemento‐enamel junction (CEJ) to the base of the alveolar bone level (ABL) was measured. The data was analyzed using SPSS.

**Results:**

Intraclass correlation analysis (ICC) revealed a moderate degree of reliability between CAL and CEJ to ABL measurements. The average ICC was 0.68 with a 95% confidence interval of 0.53–0.77 (*p* < .001) indicating moderate to good reliability. Comparing the types of teeth, the central incisors, particularly the lower central incisors showed the highest ICC values (ICC: 0.822, CI: 0.77–0.86) indicating good reliability while the premolar and molars showed poor to moderate agreement (Maxillary premolars ICC: 0.464, CI: −0.18–0.74; maxillary first molar ICC: 0.516, CI: −0.154–0.772; mandibular first premolar ICC: 0.662, CI: 0.269–0.782; mandibular first molar ICC: 0.625, CI: 0.31–0.82). A moderate correlation existed between the radiographic and the clinical assessments (***r*** = 0.5, *p* < .001).

**Conclusion:**

Despite the fact that significant varying levels of reliability has been found between CAL and radiographic bone level, both the clinical and radiographic examinations should be performed for the accuracy of diagnosis.

## 1 INTRODUCTION

Periodontitis, a disease of the supporting structures of the teeth is regarded as the second most prevalent oral disease globally, and is the primary cause of tooth loss in adults (Laine, Crielaard, & Loos, 2012; Vos et al., 2012). Clinical attachment loss (CAL) and radiographically assessed alveolar bone height are used to assess the loss of periodontal tissue support in periodontitis (Dietrich et al., 2019; Hoath, Wiebe, Garcia Fulle De Owen, Giannelis, & Larjava, 2016; Papapanou et al., 2018) and to monitor disease progression and the effect of therapy on the periodontium (Goodson, Haffajee, & Socransky, 1984; Hossain, Fageeh, & Elagib, 2013). Differences exist between the diagnostic methodologies concerning the relationship between the measurement and the position of the apical front of the lesion (Papapanou & Wennström, 1989).

CAL represents the extent of periodontal support that has been lost around a tooth and is measured with the periodontal probe as the distance from the cemento‐enamel junction (CEJ) to the base of the pocket (Highfield, 2009; Hughes, Seymour, Turner, Shahdad, & Nohl, 2012). Alveolar bone loss provides a good estimate of the overall attachment loss of the supporting structures (Kiliç, Efeoglu, Yilmaz, & Orgun, 1998). Currently, conventional and digitized periapical, bitewing, and panoramic radiographs (DPT) are used as an adjunct to clinical assessment in diagnosing periodontitis (Christiaens et al., 2018; Goodson et al., 1984). The most frequent radiographs to identify alterations in alveolar bone are periapical (PA) and bitewing radiographs (Scarfe, Azevedo, Pinheiro, Priaminiarti, & Sales, 2017). However, it is known that the panoramic radiographic technique has a limited capacity to determine and diagnose bony defects due to a lack of detail in panoramic radiographs. Small losses in the height of the alveolar bone crest should be cautiously evaluated, as they may be overestimated (Semenoff et al., 2011).

According to the literature, few studies have been done to study the relationship between CAL and alveolar bone level with inconclusive results (Esmaeli et al., 2012; Goodson et al., 1984; Machtei, Hausmann, Grossi, Dunford, & Genco, 1997; Mann, Pettigrew, Beideman, Green, & Ship, 1985; Papapanou & Wennström, 1989). Also, different statistics have been used to measure the associations. Pearson correlation coefficient, Bland–Altman plots and paired *t*‐tests have been used to evaluate the reliability in these studies which are considered as nonideal measures of reliability (Koo & Li, 2016) as they either measure correlation or analyze agreement alone. The Intraclass Correlation Coefficient (ICC) is a more appropriate measure of reliability reflecting both the degree of correlation and agreement between the measurements (Koo & Li, 2016; Stemler & Tsai, 2008; Zaki, Bulgiba, Nordin, & Ismail, 2013). However, the literature available related to the ICC analysis between CAL and radiographic alveolar bone level is scarce.

The aim of the current study was to investigate the agreement between CAL with radiographically assessed bone level in teeth affected by periodontitis. According to our knowledge, this was the first study investigating the reliability between the two measurements using ICC analysis.

## 2 MATERIALS AND METHODS

A retrospective cross‐sectional study was designed to determine the relationship between CAL and radiographic bone level using a consecutive sampling technique from the electronic records of the patients diagnosed with established periodontitis from January 2018 to July 2019. The sample size was estimated based on previously reported values of mean and *SD* (Papapanou & Wennström, 1989) which reported a pooled standard deviation of 1.36 units. Using the formula for comparison of two means estimation, a total sample of 880 sites was recommended for this study. The study has 87% power to detect the expected effect size of 0.2 mm. Our study received scientific and ethical approval from the Institutional Review Board at King Abdullah International Medical Research Centre (KAIMRC), Riyadh (# SP19/449/R).

The inclusion criteria for the subjects were the comprehensive periodontal examination information of patients diagnosed with established periodontitis entered in the electronic patient records and the presence of diagnostic quality radiographs including full mouth PA and DPT radiographs covering the entire dentition. The selected age range included subjects from 25 to 60 years who are either healthy or with a controlled systemic condition having all anterior and posterior teeth present. The exclusion criteria included radiographs performed in different dates, absence of at least one adjacent tooth, unsatisfactory positioning of the tooth in the dental arch, ill‐defined CEJ and patients with gingival enlargement or limited mouth opening.

Records of CAL and radiographs of the mesial and distal sites of the maxillary and mandibular central incisors, first premolars and first molars were obtained from the patient records. The clinical measurements obtained from each file were recorded using an UNC‐15 periodontal probe for all tooth surfaces and rounded to the nearest 0.5 mm from the CEJ to the depth of the sulcus.

**FIGURE 1:**
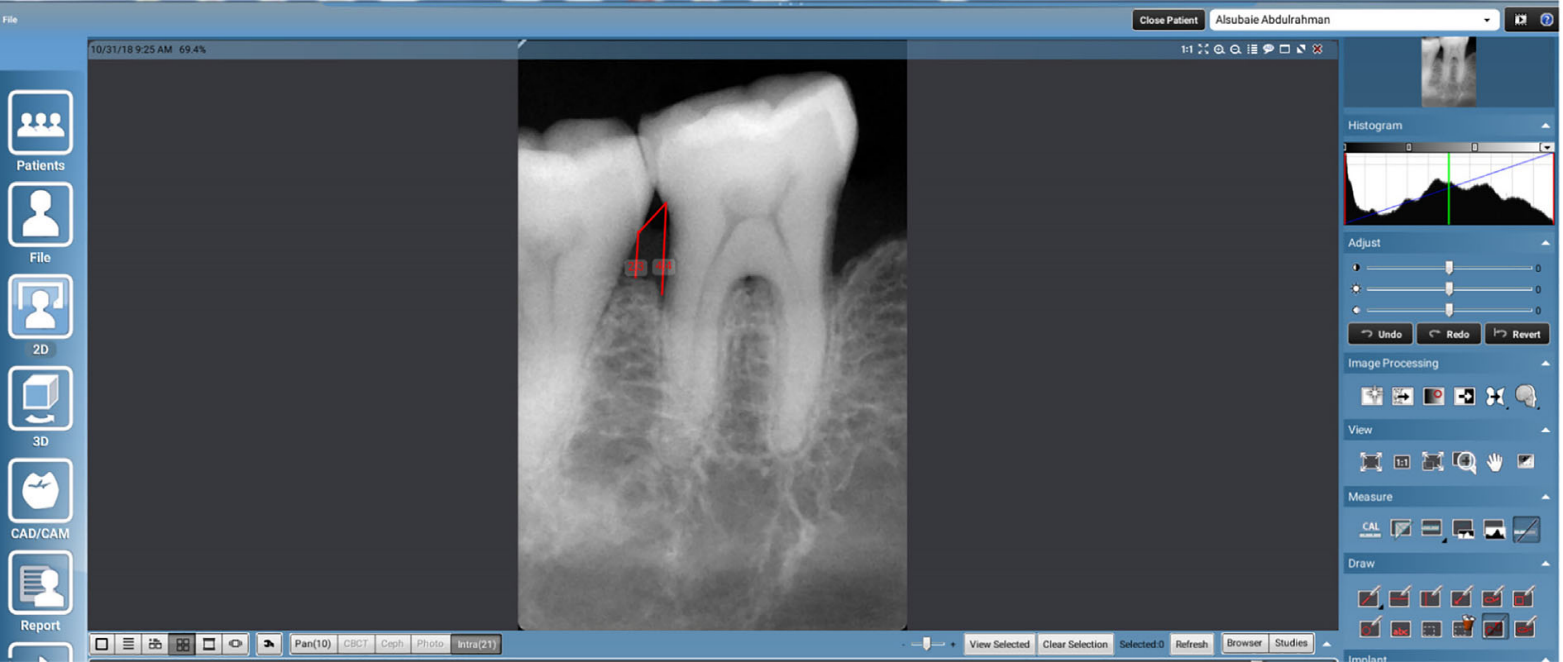
Measurement of alveolar bone level in millemeters (the distance from cemento‐enamel junction to apical part of alveolar crest) at distal side of mandibular molar

Radiographic assessment of periodontitis sites was done using the available records of periapical radiographs taken using the paralleling technique with film holders to ensure standardization (Parallel technique (Kodak Ultraspeed Dental Film, Eastman Kodak, Rochester, NY) with a Siemens Heliodent MD model X1744 (Sirona Dental Systems, GmbH D‐64625, Bensheim, Germany) X‐ray machine set to 70 kV and 7 mA) and dental panoramic tomogram (Digital panoramic radiography (Morita Veraview IC5, J. Morita MFG. Corp., Kyoto, Japan) in the Planmeca Romexis software management system). The software included a digital ruler that assisted with the measurement of the distance from the apical part of alveolar crest (AC) to the CEJ in millimetres to determine the amount of bone loss in the interproximal area (Figure 1). All the measurements were performed independently by two independent examiners.

From the dental panoramic tomogram, the linear measurement of bone loss in the interproximal area in millimetres was taken for the first premolar and first molar of the mandible and the first molar of the maxilla in the posterior teeth region.

Also, records of the percentage of alveolar bone breakdown was measured for the teeth selected in the sample in the periapical radiographs as the percentage bone loss relative to the root length. This percentage was categorized into stages, namely <15% bone loss as stage I, 15%–33% bone loss as stage II and >33% as stage III. This was then compared with the corresponding stages of CAL (Stage I (1–2 mm CAL), stage II (3–4 mm CAL) and stage III (>5 mm of CAL) (Tonetti, Greenwell, & Kornman, 2018).

The presence or absence of periodontal widening was also recorded as evident in the selected teeth from the periapical radiographs. Our definition of periodontal widening included widening near the coronal portion of the root or in the periapical region, on one or both sides of the root. This was compared with the severity of the CAL in the records designated as mild, moderate and severe. All of these measurements were performed independently by two examiners. The clinical and radiographic data were collected, tabulated and statistically analyzed using Statistical Package for Social Sciences (IBM‐SPSS) program version 23.

## 3 RESULTS

A total of 880 tooth sites were evaluated. The descriptive statistics are described in Table 1.

**TABLE 1:** Descriptive statistics

Gender	Male: 69 Female: 35
Age (years)	Mean = 53 ± 10.7 Min 35 Max 76
Sites	880
CAL	5.60 ± 2.06
CEJ‐ABL:	4.70 ± 2.04

The ICC analysis was performed to assess the reliability between the CAL and alveolar bone level in the PA and DPT (Table 2). The ICC analysis revealed a moderate degree of reliability between CAL and CEJ to ABL measurements in the periapical radiographs. The average ICC was 0.68 with a 95% confidence interval from 0.53–0.77 (*p* < .001) indicating a moderate to good reliability. Comparing the individual teeth, the central incisors, particularly the lower central incisors had the highest ICC values (ICC: 0.822, CI: 0.77–0.86) indicating good reliability while the premolar and molars showed poor to moderate reliability (Maxillary premolars ICC: 0.464, CI: −0.18–0.74; maxillary first molar ICC: 0.516, CI: −0.154–0.772; mandibular first premolar ICC: 0.662, CI: 0.269–0.782; mandibular first molar ICC: 0.625, CI: 0.31–0.82). Comparatively, the DPT showed a poor ICC estimate (Table 2). The inter‐examiner reliability was excellent (ICC≥0.998; *p* <.001).

**TABLE 2:** Intraclass Correlation Coefficient (ICC) analysis between clinical attachment level (CAL) and bone loss

Comparison	Arch	Tooth	ICC	Confidence interval
CAL and bone loss in periapical radiograph	Maxilla	Central incisor	0.735	0.597–0.822
		First premolar	0.464	−0.18– 0741
		First molar	0.516	−0.154– 0.772
	Mandible	Central incisor	0.822	0.774–0.860
		First premolar	0.622	0.269–0.782
		First molar	0.625	0.31–0.824
CAL and bone loss in dental panoramic radiograph	Maxilla	First molar	0.38	−0.12–0.66
	Mandible	First premolar	0.28	−0.05–0.50
		First molar	0.546	0.08–0.75

The correlation analysis performed between CAL and CEJ to the base of the alveolar bone in periapical radiographs showed a statistically significant moderate positive correlation (***r*** = 0.566, *p* < .001). The results of the correlation for the individual teeth are summarized in Table 3. However, the correlation between CAL and bone level in DPT showed a low positive correlation (***r*** = 0.355, *p* < .001) (Table 3).

**TABLE 3:** Pearson correlation analysis between clinical attachment level (CAL) and bone loss

Comparison	Tooth	Correlation	*p‐value*
CAL and ABL in periapical radiographs	General	0.57	*p*<.001
	Maxillary central incisor	0.62	*p*<.001
	Maxillary first premolar	0.55	*p*<.001
	Maxillary first molar	0.55	*p*<.001
	Mandibular central incisor	0.70	*p*<.001
	Mandibular first premolar	0.55	*p*<.001
	Mandibular first molar	0.61	*p*<.001
CAL and ABL in dental panoramic radiographs	General	0.36	*p*<.001
	Maxillary first molar	0.35	*p*<.001
	Mandibular first premolar	0.35	*p*<.001
	Mandibular first molar	0.52	*p*<.001

Abbreviation: ABL, alveolar bone level.

The stages of CAL and stages of bone loss correlated well with a strong positive relationship (***r*** = 0.50, *p* < .001) between the two variables in periapical radiographs whereas in DPT, a moderate positive relationship (***r*** = 0.36, *p* < .001) was shown. Agreement of comparison among the raters was almost perfect, with a kappa = 0.95 (0.93–0.97).

The agreement in relation to the stages of bone loss versus stages of CAL was fair (kappa = 0.22). The lowest agreement was for maxillary first premolar, maxillary molar and mandibular molar (kappa = 0.12–0.16); the highest agreement was for the maxillary and mandibular central incisors (kappa = 0.35). With regard to the DPT, the kappa analysis revealed none to slight agreement (kappa = 0.128).

A chi‐square test of independence showed that there was a significant association between severity of CAL and PDL widening in the periapical radiographs, *X* ^2^ (2, *N* = 874) = 23.67, *p* < .001.

## 4 DISCUSSION

Our findings from the ICC analysis demonstrated a moderate degree of reliability between CAL and the distance between the CEJ to the base of the alveolar bone in the periapical radiographs. The lower central incisors showed the highest ICC values indicating a good reliability, followed by the maxillary central incisors. All the other teeth showed none to slight agreement. With regard to the comparison of CAL with alveolar bone level in the DPT, a poor agreement was revealed. This finding is supported in literature reporting that periapical radiography had a higher accuracy and precision compared to panoramic radiography in detecting periodontal osseous destruction (Akesson, Hakansson, & Rohlin, 1992; Pepelassi & Diamanti‐Kipioti, 1997).

Historically, Pearson correlation coefficient, Bland–Altman plots and paired *t* tests have been used to evaluate reliability (Machtei et al., 1997; Papapanou & Wennström, 1989; Zhang, Rajani, & Wang, 2018). However, each of these tests have limitations, the paired *t* test and Bland–Altman plot are used for analysing agreement, the Pearson correlation coefficient measures only correlation. These are non‐ideal measures of reliability (Koo & Li, 2016). Though, correlation and agreement, measure the strength of association between variables of interest, they are conceptually distinct (Jinyuan, Wan, Guanqin, Yin, & Changyong, 2016). Correlation measures the association between two continuous outcomes when the relationship between the variables is linear and correlation of variables can be assessed for variables that measure completely different constructs. Assessing agreement between variables assumes that the variables measure the same construct (Jinyuan et al., 2016). Similar to correlation, agreement also assesses the relationships between outcomes of interest, but, emphasizes on the degree of concordance in the results between two or more assessments of the variable of interest (Jinyuan et al., 2016).

ICC is considered a more acceptable measure of reliability that reflects both the degree of correlation and agreement between measurements (Koo & Li, 2016). To our knowledge, this was the first study that investigated the agreement between clinical attachment loss and alveolar bone level in periapical and DPT radiographs using ICC. Overall, we found almost perfect interrater reliability.

The findings of our study in terms of the correlation analysis, revealed a moderate positive correlation between the radiographic (periapical) and the clinical assessments (***r*** = 0.5, *p* < .001) which was similar to Zhang et al. (2018) and Machtei et al. (1997) reporting an overall moderate positive correlation between CAL and bone height measurement (***r*** = 0.55 and ***r*** = 0.73, *p* < .001). Our findings were dissimilar to that of Papapanou and Wennström (1989) who found a strong positive correlation between the clinical and bone height measurements and periapical radiographs (***r*** = 0.8, *p* < .001). Papapanou et al. also revealed that there was no difference between the two variables irrespective of the tooth type and tooth surface but the correlation was poor at sites with severe periodontal tissue breakdown. It should be noted that the study was conducted three decades ago and with the development of recent advances in the field of imaging and technology, new research is required. Another possible reason for the disagreement in findings could be the differences in reference points for the measurements. The Papapanou et al. study used the distance between the CEJ and the most coronal level of the alveolar bone in contrast to our study where the most apical point of the bone defect was used.

Our study is also contrary to Mann et al. (1985) . However, the study made the comparison of the clinical measurements with the bitewing radiographs. Mann et al. showed a poor agreement between the clinical and radiographic assessments and included paediatric patients (12 years to 16 years). They considered only the presence and absence of a pathological crevice in the clinical assessment and presence and absence of alveolar bone loss. .Diab et al. showed a very strong correlation between the two variables. However, they had a small sample size and estimated only the posterior teeth using bitewing radiographs. No studies were found comparing the relationship between the severities of CAL and alveolar bone loss. This was the first study to investigate the relationship between the different stages of CAL and alveolar bone loss.

Our study demonstrated a significant association between the severity of CAL and PDL widening in the periapical radiographs. It has been reported in the literature that periodontal disease and periodontitis are the infectious causes of PDL widening (Mortazavi & Baharvand, 2016) and it is the periodontal pathogens or the spread of infection that promotes widening of the periodontal ligament. However, the association of CAL with PDL widening has not been proven scientifically. PDL widening is detected in the early stages of periodontitis when bone loss progresses down the root of the teeth in association with a deep periodontal pocket (Kumar, 2014). Other evidence includes widening of the PDL at the apex or inter‐radicular area in periodontal disease (White & Pharoah, 2014).

## 5 CONCLUSION

Within the limits of this study, the lower central incisors showed the highest degree of reliability between CAL and radiographic bone levels. Anterior teeth revealed good reliability than the posterior teeth. Considering the significant varying levels of reliability between the CAL and radiographic bone levels in periapical radiographs, a combination of clinical and radiographic examinations should be performed for the accuracy of diagnosis. Further prospective studies should be conducted to support the findings and strengthen the evidence.
